# The Effect of Aromatherapy Alone or in Combination with Massage on Dysmenorrhea: A Systematic Review and Meta-analysis

**DOI:** 10.1055/s-0041-1740210

**Published:** 2021-12-21

**Authors:** Mona Najaf Najafi, Neshat Najaf Najafi, Farzaneh Rashidi Fakari, Somayeh Moeindarbary, Fatemeh Abdi, Zeinab Sadat Hoseini, Masumeh Ghazanfarpour

**Affiliations:** 1 Clinical Research Development Unit, Mashhad University of Medical Sciences, Imam Reza Hospital, Mashhad, Iran; 2 Clinical Research Development Unit, Mashhad University of Medical Sciences, Imam Reza Hospital, Mashhad, Iran; 3 Department of Midwifery and Reproductive Health, School of Medicine, North Khorasan University of Medical Sciences, Bojnurd, Iran; 4 Department of Obstetrics and Gynecology, Neonatal and Maternal Research Center, Mashhad University of Medical Sciences, Mashhad, Iran; 5 School of Nursing and Midwifery, Alborz University of Medical Sciences, Karaj, Iran; 6 Faculty of Medicine, Islamic Azad University of Mashhad, Mashhad, Iran; 7 Student Research Committee, Kerman University of Medical Sciences, Kerman, Iran

**Keywords:** dysmenorrhea, herbal medicine, aroma oil, aromatherapy, dismenorreia, fitoterapia, óleo de aroma, aromaterapia

## Abstract

**Objective** The aim of the present systematic review meta-analysis is *to assess the effect of olfactory stimulation on reducing dysmenorrhea.*

**Methods** Systematic search was conducted in several databases, such as PubMed, Web of Science, Cochrane, and Scopus, to identify relevant research up to October 26, 2019. The identified studies were evaluated based on a modified Jadad scale. The intervention involves aromatherapy alone or in combination with essential oils. There was no restriction for the control group such as a placebo group or other common treatments. The Comprehensive Meta-Analysis Version 2 (Bio stat, Englewood, NJ, USA) was used for meta-analysis. Cochran's Q and I2 tests were utilized.

**Results** The findings of our meta-analysis, which contained 13 trials (15 data), showed that dysmenorrhea decreased significantly in the group receiving aromatherapy with herbal compared with the control group (standardized mean difference [SMD] = -0.795; 95% confidence interval [CI]: -0.922 to- 0.667; 17 trials O < 0.001); heterogeneity; I2 = 19.47%; *p* = 0.236). In addition, four studies with insufficient data were not included in our meta-analysis. The results of all studies suggested that aromatherapy with herbal medicine group compared with control group is effective.

**Conclusion** Aromatherapy with herbal medicine decreased dysmenorrhea. This treatment was particularly effective when aroma oil was combined with massage or when a mixture of aroma oil was used for the treatment of dysmenorrhea.

## Resumo

**Objetivo** O objetivo desta revisão sistemática-metanálise é avaliar o efeito da estimulação olfatória na redução da dismenorreia.

**Métodos** Pesquisa sistemática foi realizada em várias bases de dados, como PubMed, Web of Science, Cochrane e Scopus para identificar pesquisas relevantes até 26 de outubro de 2019. Os estudos identificados foram avaliados com base em uma escala de Jadad modificada. A intervenção envolve aromaterapia sozinha ou em combinação com óleos essenciais. Não houve restrição para o grupo de controle, como um grupo de placebo ou outros tratamentos comuns. O *Comprehensive Meta-Analysis Version 2* (Bio stat, Englewood, NJ, EUA) foi usado para meta-análise. Os testes Q e I2 de Cochran foram utilizados.

**Resultados** Os resultados da nossa meta-análise, que continha 13 ensaios (15 dados), mostraram que a dismenorreia diminuiu significativamente no grupo que recebeu aromaterapia com ervas em comparação com o grupo de controle (diferença média padronizada [DMP] = -0,795; intervalo de confiança [IC] de 95%: -0,922 a- 0,667; 17 ensaios O < 0,001); heterogeneidade; I2 = 19,47%; *p* = 0,236). Além disso, quatro estudos com dados insuficientes não foram incluídos em nossa meta-análise. Os resultados de todos os estudos sugeriram que a aromaterapia com o grupo de fitoterápicos em comparação com o grupo de controle é eficaz.

**Conclusão** A aromaterapia com fitoterapia diminuiu a dismenorreia. Este tratamento foi particularmente eficaz quando o óleo aromático foi combinado com massagem ou quando uma mistura de óleo aromático foi usada para o tratamento da dismenorreia.

## Introduction

Menstrual pain, a common complaint expressed by ∼ 25 to 97% of women, can influence the quality of life in women. 1 2 Dysmenorrhea refers to painful menstruation associated with several adverse effects, including nausea and vomiting, back pain, fatigue, and abdominal cramps. 3 The severest pain is experienced in the first day of menstruation, and it takes a downward turn in the remaining days. Moreover, the pelvic pain associated with menstrual cycle is caused by the secondary dysmenorrhea coupled with pelvic discomforts, which is common during reproductive age and ovulation. Dysmenorrhea is reported to be the most prevalent cause of absenteeism in female students and employees. 4

The incidence of dysmenorrhea is reported to be between 28 and 71.7%, but these figures vary worldwide. 5

The primary causes of dysmenorrhea are uterine contraction, vasoconstriction, inflammation, and the release of inflammatory mediators. Diminished progesterone level in the late luteal phase leads to the activation of cyclooxygenase and the biosynthesis of prostaglandin. Elevated prostaglandin production increases uterine tone and contractions, followed by dysmenorrhea. 6 It has been shown that dysmenorrhea is a major disease affecting physical, social, and psychological parameters including social, emotional, and mental health. 7 Dysmenorrhea may interfere with sports activities, disrupt communication with family and friends, and lower the quality of life. 8 9

Drug therapy is commonly used for the management of dysmenorrhea, especially anti-inflammatory agents, and is the first line in the treatment of primary dysmenorrhea. Nevertheless, these drugs present documented unwanted complications, including drowsiness, headache and dyspepsia, 10 nausea, vomiting, rashes, nervous disorders, emotional disturbances, bleeding tendency, metabolic disorders, and many more, including cancers and death. 11 Another therapeutic regimen for dysmenorrhea involves the use of hormonal agents, such as oral contraceptive. 12 By reducing prostaglandins, these agents have a soothing effect on dysmenorrhea. Also, hormonal therapy is recommended when treatment with non-steroidal antiinflammatory drugs is ineffective or when contraceptive methods are preferred. 13 Despite the effect of these agents on pain reduction, irregular bleeding is a major factor that provokes women's dissatisfaction with hormonal treatment. 14 Other side effects of this therapy include headache, nausea, mood changes, and weight gain. 13 Medicinal plants have received growing attention for the attenuation of dysmenorrhea, particularly due to the reluctance of young women to hormonal drug administration, the complications of chemical medications, and the high cost of raw materials. 15 However, most of these treatments cannot be administered by nurses, and routine drugs exhibit adverse effects or produce short-term effects. 16 Despite the effectives of these drugs in alleviating premenstrual syndrome, women believe that these symptoms can be managed without using any drug or prefer using traditional and alternative techniques. 17 There are various complementary and alternative medicine (CAM) methods used for menstrual pain relief, such as behavioral interventions, acupuncture, herbal medicine, transcutaneous electrical nerve stimulation (TENS), omega-3 fatty acids, vitamin pills, 18 and aromatherapy. As a safe therapeutic technique, aromatherapy relies on plant-based essential oils (Buckle, 2001), 19 including melissa, lavender, and eucalyptus.

Many clinical studies assessed the effect of aromatherapy on dysmenorrhea. Three studies showed a significant reduction of pain in patients receiving rose essential oil in comparison with a control group. 3 20 21 Studies showed that lavender was effective both as monotherapy 10 15 22 and in combination with another oil. 23 24 25 26 Peppermint, 27 rosemary, 22 and geranium essential oils 28 have shown to be effective in clinical studies. Pervious meta-analyses have illustrated the effectiveness of aromatherapy as a complementary method in reducing dysmenorrhea. 29 30 According to the results of a meta-analysis, the use of essential oil in combination with massage was more effective than the control group. However, the study did not clarify whether a mixture of aroma oils or a single oil produced greater efficacy. Recently, new trials with a well-designed methodology have been published; therefore, it is necessary to update the meta-analysis. The aim of the present systematic review and meta-analysis is to assess the effect of olfactory stimulation in the treatment of dysmenorrhea.

## Methods

A comprehensive systematic search without any language restriction was conducted in several databases such as PubMed, Web of Science, Cochrane, and Scopus using the follwing keywords: ( *dysmenorrhea* ) OR ( *menstrual cramp* ) OR ( *menstrual pain* *) OR ( *pain* *, *menstrual* ) OR (painful menstruation*) OR ( *menstruation* *, *painful* )) AND ( *aromatherapy* ) OR ( *Aroma* *) OR ( *aromatic therapy* ) OR ( *fragrance* ) OR ( *fragrant oil* ∗) OR ( *scent* ) OR ( *massage therapy* ) OR ( *medical massage* ) OR ( *massage* ). The relevant studies were investigated up to October 26, 2019. All terms were searched in the title, abstract, and key words of the articles. In addition, the bibliography of the selected articles was manually searched to identify other relevant studies missed in the electronic search. Gray literature was not included in the present review.

We examined clinical trials on girls and women of childbearing age (18–45 years old) who experienced menstrual pain. The intervention had to involve aromatherapy alone or combined with essential oils in the form of inhalation or massage. There was no restriction regarding the control group; therefore, no treatment, a placebo group, or other common treatments for dysmenorrhea were considered. The severity of pain should be assessed by a valid self-reported instrument, such as a numerical rating scale (NRS) or visual analog scale (VAS).

**Fig. 1:**
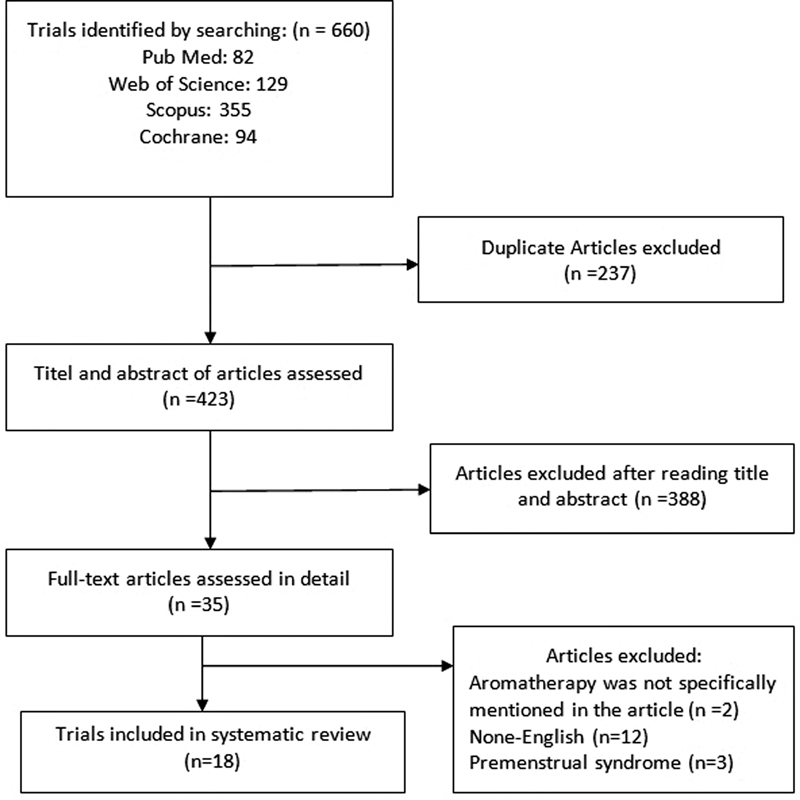
Provides flowchart of study selection for the review.

The databases were searched by two independent researchers, and duplicate papers or papers that did not meet the inclusion criteria were excluded. The selection process is shown in Fig. 1 . Observational studies, reviews, letter to the editor and case reports were excluded. Additionally, publications written in a language other than English were also excluded. Three English abstracts (full texts were available in Farsi) were explored. 20 21 23 28 31 32 33 34 35 36 37 38 39 40 41 42 43 44 45 46 47 48

Two authors assessed the quality of the selected studies according to the Modified Jadad Scale for Randomized Controlled Trials. This assessment tool comprises eight specific items, including a description of randomization, appropriate randomization method, blinding description, appropriate blinding method, description of withdrawals and dropouts, description of inclusion and exclusion criteria, description of adverse effects, assessment method, and the description of statistical analysis. The scores range from 0 to 8, with higher scores indicating a higher quality. A score below 4 indicates low quality.

The data was extracted by two researchers. Any disagreements were settled through consensus and arbitration of a third person.

The selected studies were reviewed, and the following data was extracted by two authors: first author's name, year of study, place of study, type of study design, participants, the age of participants, intervention and control groups, the number of participants in the intervention and control/placebo groups, duration of study, sample dropout rate (%), measurement method, minimal primary dysmenorrhea for inclusion in the study, and the primary outcome. The primary outcome was pain, which was measured by VAS score, numerical rating scales, or other validated instruments.

In the present study, the Comprehensive Meta-Analysis Version 2 software (Bio stat, Englewood, NJ, USA) was used for the meta-analysis. We used Cochran's Q and I ^2^ tests to evaluate heterogeneity and homogeneity, respectively. If the test result of Cochran's Q was *p* < 0.05, the heterogeneity of studies was confirmed. Moreover, I ^2^ values of 25%, 50%, or 75% exhibited low, moderate, or high heterogeneity, respectively. If heterogeneity was confirmed, we used the random effects model for the analysis. To present treatment effects, we used a forest plot that demonstrated the effect size, and a confidence interval of 95%. The Begg rank correlation and the Egger intercept tests were employed to evaluate publication bias. We examined a funnel plot for checking publication bias, and the number of missing studies was computed by the Duval and Tweedie Trim and Fill program.

## Results

The review of literature based on the above-mentioned search strategy yielded 660 articles, 237 of which were duplicate and, therefore, removed. Subsequently, we reviewed the titles and abstracts of studies, excluding 388 articles that had irrelevant titles or abstracts. In the end, 35 full-text articles were assessed completely, of which 17 papers were excluded. Aromatherapy was not specifically stated in two article, 4 32 12 articles were not in English, 18 33 34 35 36 37 38 39 40 41 42 43 and the symptoms of premenstrual syndrome had been examined in three papers. 17 44 45 Finally, 18 clinical trials undertaken until October 26, 2019 were included in this systematic review. The flowchart identifying the included articles is shown in Fig. 1 .

These studies are listed in Table 1 . The trials consisted of a total of 1,677 patients. All of these studies targeted adult patients under 35 years of age. The essential oils consisted of lavender essence (seven studies), rose essential oil (three studies), rosemary oil (one study), ginger oil (one study), Geranium essential oil (one study), and peppermint oil (one study) as well as a combination of several essential oils (seven studies).

**Table 1:** The quality assessment of the selected articles

	Registration code	Randomization mention	Random sequence generation	Blinding of participants and personnel	Blinding of outcome assessment	Description of withdrawals and dropouts	Clear description of the inclusion and exclusion criteria	Description of the method used to assess adverse effects	The method of statistical analysis	Total score
Apay et al. (2012) 22	−	1	0	1	0	1	1	1	1	6
Amiri Farahani et al. (2012) 24	IRCT138902153869N13869	1	0	1	0	1	1	1	1	6
Ataollahi et al. (2015) 21	IRCT201311216807N10	1	1	1	1	1	1	1	1	8
Azima et al. (2015) 10	IRCT2013022611945N2	1	1	0	1	1	1	0	1	6
Bakhtshirin et al. (2015) 15	−	1	0	1	1	1	1	0	1	6
Beiranvand et al. (2015) 31	IRCT201310297697N2	1	1	1	1	1	1	0	1	7
Davari et al. (2014) 23	−	1	0	1	1	1	1	1	1	7
Raisi Dehkordi et al. (2014) 47	IRCT201105086412N1	1	1	1	1	1	1	1	1	8
Kim et al. (2011) 18	−	0	0	0	0	1	1	1	1	4
Marzouk et al. (2013) 25	−	1	1	1	1	1	1	1	1	8
Ou et al. (2012) 26	−	1	1	1	1	1	1	0	1	7
Uysal et al. (2016) 3	−	1	1	0	0	1	1	0	1	5
Hur et al. (2012) 1	−	0	0	0	0	1	1	1	1	4
Nikjou et al. (2016) 48	IRCT201470616252N2	1	1	1	1	1	1	1	1	8
Rizk (2013) 27	−	1	0	1	0	1	1	0	1	5
Sadeghi Aval Shahr et al. (2015) 20	−	1	0	1	0	1	1	1	1	6
Sajjadi et al. (2018) 28	IRCT2017013132329N1	1	0	1	1	1	1	0	1	6
Han et al. (2006) 16	−	1	0	1	0	1	1	1	1	6

The selected studies were evaluated based on a modified Jadad scale. The scale includes 8 items. Most of the articles (17 papers) had high quality (a Jadad score of 5–8), and only one was of low quality (a Jadad score of 4). In 8 papers, suitable randomization methods were described, and patients were blind to the randomization in 14 studies. The rate of withdrawals and dropouts had been reported in all studies, whereas the assessment of adverse effects was only discussed in 11 articles. The statistical methods and inclusion criteria were adequately described in all RCTs. The quality assessment of the selected articles is depicted in Table 1 . A comprehensive list of the selected studies' characteristics is outlined in Table 2 .

**Table 2:** Detailed characteristics of the selected studies

Study (year)	Type of study	Participants	Age (mean)	Intervention (n)	Control (n)	Duration of study	Sample drop (%)	Measurement method	Inclusion	outcome
Apay et al. (2012) 22	Quasi-experimental crossover	Midwifery/nursing students	20	Lavender/ abd. Massage, 1st day of menses, 15 minutes, 1 time ( *n* = 44)	Placebo/abd. Massage 1st day of menses, 15 minutes, 1 time	3 cycles	0	VAS-100 points	VAS > 60	Massage is effective. The effect of aromatherapy massage is higher than placebo massage ( *p* < 0.001).
Amiri Farahani et al. (2012) 24	RCT	Medical university students	21	Massage + mixture of lavender/peppermint essential oil ( *n* = 36) a week before menses up to the presence of pain, one time, 15 minutes, daily	1.almond oil ( *n* = 36) 2.massage alone ( *n* = 36) a week before menses up to the presence of pain, one time, 15 minutes, daily	2 cycles	reported	VAS-10points	multidimensional spoken criteria = 2 or 3	Severity of dysmenorrhea decreased in massage and aromatherapy groups( *p* = 0.014)
Ataollahi et al. (2015) 21	RCT	Medical student	21	Rosaceous extract ( *n* = 55), First 3 days of menses, two times daily	Placebo ( *n* = 55) First 3 days of menses, two times daily	2 cycles	0	VAS-10points	Intermediate/ sever pain	Severity of dysmenorrhea decreased in both groups, but this reduction was more significant in the Rosaceous group ( *p* < 0.001).
Azima et al. (2015) 46	RCT	Non-medical university students	21	Lavender/massage ( *n* = 34) First 2 days of menses, one time daily	1) Reflexology ( *n* = 34), 20 minutes daily, 10 days before menses 2) Control ( *n* = 34)	2 cycles	0	VAS-10points	VAS > 5	pain reduction was more in the lavender/ massage group ( *p* < 0.001)
Bakhtshirin et al. (2015) 15	RCT, cross over	Midwifery/nursing students	20	Lavender/ massage ( *n* = 40)	placebo/ massage ( *n* = 40)	2 cycles	0	VAS-10points	VAS > 6	A significant pain reduction in lavender/massage in comparison with placebo/massage.
Beiranvand et al. (2015) 31	RCT cross over	Students	21	Lavender/ 48 hours before and after menstruation/ 15 minutes twice a day ( *n* = 30)	Placebo (almond oil)/ 48 hours before and after menstruation/ 15 minutes twice a day ( *n* = 30)	2 cycles	reported	VAS-10points	VAS > 5	A significant pain reduction in lavender/massage in comparison with placebo/massage ( *p* < 0.001).
Davari et al. (2014) 23	RCT	Students	22	Rosemary ( *n* = 30), Lavender ( *n* = 30), rosemary + lavender ( *n* = 30), first 3 days of menses 15 minutes, twice a day	mefenamic acid ( *n* = 30), placebo ( *n* = 30)	2 cycles	reported	VAS-10points	MDQ	significant reductions in pain in rosemary ( *p* < 0.001), lavender, both, and mefenamic acid ( *p* < 0.01) compared with placebo.
Raisi Dehkordi et al. (2014) 47	RCT Crossover	Students	20	Lavender first 3 days of menses, every 6 hours ( *n* = 48)	Placebo first 3 days of menses, every 6 hours ( *n* = 48)	2 cycles	reported	Scoring from 1 to 4 1: none, 2: mild, 3: moderate, 4: severe	VMS: 2 or 3	significant reductions in pain in lavender ( *p* < 0.001)
Kim et al. (2011) 18	Non-Random CT	Nurses	25	Aroma 1 + self-massage ( *n* = 26) twice a day, 2 days	Placebo ( *n* = 18) No treatment ( *n* = 19)	1 cycle	reported	VAS-10points	VAS > 5	significant reductions in pain ( *p* < 0.001)
Marzouk et al. (2013) 25	RCT Crossover	Nursing students	17–20	Aroma 2 + massage ( *n* = 48), once daily, 7 days before menses	Placebo+ massage ( *n* = 47), once daily, 7 days before menses	2 cycles	reported	VAS-10points	VAS > 5	significant reductions in pain
Ou et al. (2012) 26	RCT	Patients	24	Aroma 3 + massage ( *n* = 24) Once daily, 3 days	Placebo+ massage ( *n* = 24) Once daily, 3 days	1 cycle	0	VAS-10points	VAS > 5	Duration of pain significantly decreased
Uysal et al. (2016) 3	RCT	Patients admitted to the emergency unit	21	rose essential oil + diclofenac, 75 mg ampule ( *n* = 52)	Placebo + diclofenac, 75 mg ampule ( *n* = 53)	continuously spray every 10 minutes	reported	VAS-10points	VAS > 5	significant reductions in pain after 30 minute ( *p* = 0.019)
Hur et al. (2012) 1	RCT	High school students	?	Aroma 4 + massage ( *n* = 32)	acetaminophen ( *n* = 32)	1 cycle	reported	VAS-10points	VAS > 5	significant reductions in pain after 24 hour ( *p* < 0.001)
Nikjou et al. (2016) 48	RCT Triple blind	Students	19–29	Lavender ( *n* = 100) once a day, 30 minutes, 3 days	Placebo ( *n* = 100) once a day, 30 minutes, 3 days	2 cycles	0	VAS-10points	Intolerable, limits activities exclude	significant reductions in pain ( *p* < 0.001)
Rizk (2013) 27	RCT	Nursing students	17–21	Peppermint oil + massage ( *n* = 40) ginger oil + massage ( *n* = 40), once daily, 15 minutes, 5 days before menses	Placebo + massage ( *n* = 40)	2 cycles	reported	VAS-10points	Moderate or severe dysmenorrhea	Significant reductions in severity of pain in Int. groups.
Sadeghi Aval Shahr et al. (2015) 20	RCT	Students	18–35	Rose essential oil ( *n* = 25) on the first day of menses, 15 minutes	almond oil + massage ( *n* = 25) massage ( *n* = 25)	2 Cycles	reported	VAS-10points	VAS > 5	Significant reductions of pain in rose essential oil comparison to massage only ( *p* < 0.001) or almond oil +massage ( *p* < 0.05)
Sajjadi et al. (2018) 28	RCT	Students	18–35	Geranium essential oil ( *n* = 30) on the first day of menses, 15 minutes	1) Almond oil ( *n* = 30) 2) No treatment ( *n* = 30)	2 cycles	reported	VAS-10points	VAS > 5	Significant reduction in Geranium essential oil group comparison to others ( *p* < 0.001).
Han et al. (2006) 16	RCT	Students	20	Aromatherapy 5 + massage ( *n* = 25) 15 minutes on the first day of menses	1) Almond oil + massage ( *n* = 20) 2) No treatment ( *n* = 22)	2 cycles	0	VAS-10points	VAS > 6	The severity was significantly lower in the aromatherapy group than in the other two groups

Abbreviations: MDQ, mood disorder questionnaire; RCT, randomized control trial; VMS, verbal multi-dimensional scoring system.

1 Aroma: otto ( *Rosa damascena* ), clary sage ( *Salvia sclarea* ), rose geranium ( *Pelargonium graveolens* ), and ginger ( *Zingiber officinale* ) in almond, jojoba, and evening primrose oil.

2 Aroma: cinnamon, clove, rose, and lavender in a base of almond oil.

3 Aroma: lavender, clary sage, and marjoram oils, in jojoba cream.

4 Aroma: clary sage, marjoram, cinnamon, ginger, and geranium in almond oil.

5 Aroma: lavender ( *Lavandula officinalis* ), clary sage ( *Salvia sclarea* ), and rose ( *Rosa centifolia* ) in almond oil.

**Fig. 2:**
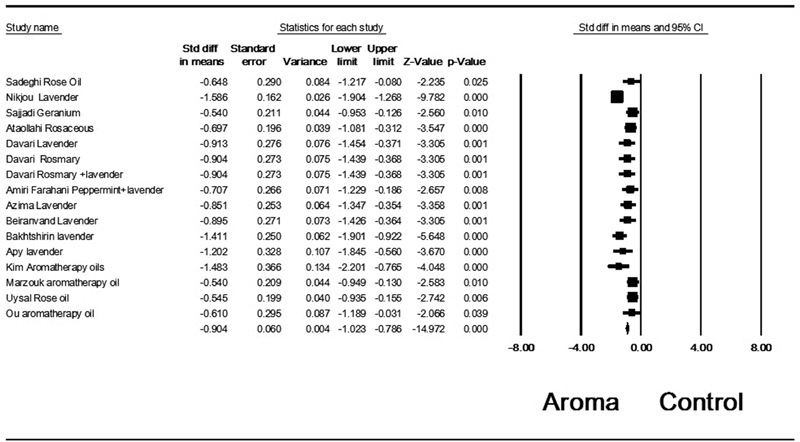
Aromatherapy effect on the severity of dysmenorrhea based on the random effects model.

**Fig. 3:**
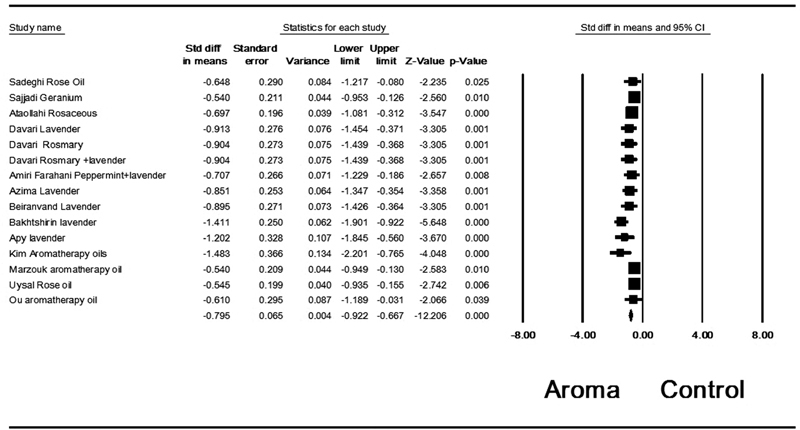
Aromatherapy effect on the severity of dysmenorrhea based on the random effects model after removal of one study which caused heterogeneity.

The standardized mean difference (SMD) of dysmenorrhea change was -0.904 (95% confidence interval CI: -1.023 to- 0.786;16 trials, *p* < 0.001) ( Fig. 2 ). However, the heterogeneity of the included studies was moderate (I ^2^ = 60.43%) and significant ( *p* = 0.003). To detect the potential source of this heterogeneity, sensitivity analyses were performed, with the results indicating that heterogeneity was mainly caused by inclusion of Nikjou et al.'s 48 study. The removal of this study caused a slight decrease in (SMD = -0.795; 95% CI: -0.922 to- 0.667; 15 trials), but the heterogeneity reached acceptable level ( *p* = 0.236; I ^2^ = 19%) ( Fig. 3 ). The results of our meta-analysis of 15 trials showed that dysmenorrhea dropped significantly in the group of aromatherapy with herbal medicine compared with the placebo. The subgroup involved the type of treatment (a combination of aroma oils versus a single type of oil, and both aroma and massage versus aroma alone). The results of the analysis of the lavender subgroup indicate higher effectiveness in the intervention than in the control group ( *p* < 0.001) ( Table 3 ). The results of subgroup analysis revealed that patients receiving a combination of massage + aromatherapy reported greater pain relief than those receiving aromatherapy alone ( *p* < 0.001). The two groups were not significantly different in the subgroup analysis comparing a mixture of aroma oils and a single oil ( *p* = 0.34).

**Table 3:** Subgroup analyses of the effect of aromatherapy in dysmenorrhea

Variable	Number of RCTs	Sample size (treatment/control)	Test of heterogeneity		*P* -value	Fixed = effect model
			*P*	I ^2^ (%)		SMD (95% CI)
**Types of treatment**						
Massage + aromatherapy	9	540	0.7	0%	< 0.001	-0.915 (-1.09 to -0.73)
Aromatherapy alone	9	729	0.13	34%	< 0.001	-0.741 (-0.89 to -0.58)
Test for subgroup difference						*p* < 0.001
**Types of treatment**						
A mixture of aroma oils	7	452	0.410	3%	< 0.001	-0.865 (-1.01 to -0.715)
Single oil	11	817	0.273	20%	< 0.001	-0.75 (-0.97 to -0.53)
Test for subgroup difference						*p* = 0.34

Abbreviations: CI, confidence interval; SMD, standardized mean difference.

Four studies with insufficient data were not included in our meta-analysis. The results of three studies 1 16 27 are presented as medians and interquartile ranges, and one study as graded severity of dysmenorrhea (mild, moderate, and severe). 47 Han et al. 16 drew a comparison between the effect of almond oil + massage and placebo with no treatment ( *n* = 22). Regression aromatherapy was associated with significant changes in the severity of dysmenorrhea (first day: β= 0.31, *p* = 0.02; second day: β= 0.33, *p* = 0.006) compared with the control and placebo groups. 16 Hur et al. 1 compared the effect of aroma (including clary sage, marjoram, cinnamon, ginger, and geranium in almond oil) + massage ( *n* = 32) to that of a control group using acetaminophen ( *n* = 32). Regression aromatherapy was strongly associated with a decrease in the severity of dysmenorrhea (β = −3.07, 95% CI −3.83 to −2.29, *t* = − 8.00, *P* < 0.001). In a 2013 study by Rizk, 27 the effects of both peppermint oil + massage ( *n* = 40) and ginger oil + massage ( *n* = 40) were compared with placebo, with the findings suggesting a significant reduction in the severity of pain. According to Raisi Dehkordi et al., 47 the ordinal logistic regression indicated that aromatherapy was significantly effective in relieving dysmenorrhea symptoms ( *p* < 0.001).

## Discussion

In this questionable pain relief mechanism, aromatherapy has been proven to influence the olfactory-hippocampal pathway, which stimulates the putative and gamma-aminobutyric acid (GABA)ergic neurons (such as cholinergic neurons), regulates the release of acetylcholine, or changes the feeling of pain. The olfactory receptors can be triggered by essential oil inhalation, which transmits signals to the brain and induces a composition of memory, thought, and emotion. Subsequently, it leads to the secretion of some internal chemicals such as endorphin and enkephalin, which can alleviate anxiety and pain, respectively. 3

The identified major compounds of rose oil are β-citronellol (14.5–47.5%), nonadecane (10.5–40.5%), geraniol (5.5–18%), and nerol and kaempferol. Rose oil has many therapeutic applications, including anticonvulsant, and analgesic and hypnotic effects. 49 Three studies assessed the effect of rose oil on dysmenorrhea. In the study of Ataollahi et al., 21 the severity of dysmenorrhea in medical students fell significantly in the rosaceous group compared with the placebo group. In the research, by Uysal et al., 3 rose essential oil + diclofenac was more effective in pain relief after 30 minutes compared with placebo + diclofenac. Sadeghi Aval Shahr et al. 20 reported a significant pain relief in the rose essential oil group compared with the massage only and the almond oil + massage groups. The essential oil of the geranium plant contains geraniol, citronellol, terpineol, and alcohols, which have anticancer, antimicrobial, antiinflammatory, analgesic, and antioxidant effects. 50 Sajjadi et al. 28 found a significant pain relief in patients receiving geranium essential oil in comparison with almond oil. The mechanism of pain relief in dysmenorrhea may be related to the analgesic effect of geranium.

Rizk 27 compared the effect of peppermint oil + massage with that of placebo, with their results suggesting a significant reduction in the severity of pain in the peppermint oil + massage group.

Peppermint oil has antispasmodic, 51 52 53 analgesic, 54 antiinflammatory, and antimicrobial effect, 55 and it can inhibit prostaglandin F2α. 56 57

The bioactive compounds of *Rosmarinus officinalis* oil significantly decreased the expression of IL-1β and TNF-α and inhibited COX-2 expression. 58 Davari et al. (2014) 23 reported a significant pain relief in the rosemary group compared with the placebo group.

Some aromatherapy textbooks suggest that two or more oils should be combined to achieve a synergic effect. 18 22 Consistent with this hypothesis, our meta-analysis revealed that a mixture of aroma oil was more effective than a single aroma oil.

Our meta-analysis shows that a combined use of both aromatherapy and massage is more effectiveness than aromatherapy alone. There is no detailed information on how to relieve pain by massage. The gate control theory of pain is one of the most influential theories which is according to in fact so that dorsal spinal horn cells acts like a gate which inhibits or facilitates transmission from the body to the brain on according to the diameters of the active peripheral fibers. There are three types of nerve fibers, A, B and C, based on the conduction velocity and the axonal diameter. The massage signals are rapidly transmitted via the sheathed A fiber, whereas pain signals are slowly transmitted via the unsheathed C fibers. The massage transfers these signals rapidly, developing pressure signals that subsequently close the pain signal gate.

According to the gate control theory, such signals encounter “nerve gates” on the spinal cord and should be cleared via these gates to travel to the brain. Other benefits of massage include the relaxation of contracted muscles, reduction of stress, and enhancement of lymph and blood circulation. 10 The effect of lavender on diminishing pain can be attributed to its sedative, antidepressant, antispasmodic, antiflatulent impact. It can also be used to treat infertility, infection, anxiety, fever, and stress. 48

Our meta-analysis, in keeping with pervious meta-analyses, illustrated that aromatherapy oil in combination with massage displayed a greater effectiveness compared with the control group. 30 According to the results of Sut and Kahyaoglu-Sut's 29 meta–analysis, RCTs decreased the risk of bias significantly in comparison with the placebo group.

The present meta-analysis has several limitations that need to be addressed. First, the power of our meta-analysis declined in various subgroups. Secondly, plants may have different therapeutic powers depending on the country or region in which the plants are harvested. 25 The absorption and metabolism of some herbs vary in different individuals. The effectiveness of herbal medicine in the treatment of dysmenorrhea may depend on the pathological source of dysmenorrhea. 48 Third, different massage methods and techniques, and factors such as massage site and pain severity may have influenced our meta-analysis. The severity of dysmenorrhea reported in each study before treatment was different. For example, Ataollahi et al. 21 and Apay et al. 22 reported a mean severity of 4.98 and 82. This may be due to several reasons. The highest level of menstrual pain appeared on the first day of menstruation and then subsided. 18 It has also been suggested that the perception and tolerance of pain may be influenced by various factors such as culture, society, and lifestyle. 15 Lastly, in some studies, the subjects were students residing in a dormitory. It may be difficult to provide suitable conditions for the application of aromatherapy in a dormitory.

## Conclusion

Aromatherapy with herbal medicine was found to alleviate dysmenorrhea. Also, a combination of oil and massage had a greater effect compared with the control group. Aromatherapy is recommended to women suffering from dysmenorrhea, especially to women who are reluctant to use hormonal drugs or concerned about the complications or high costs of chemical medications and, therefore, prefer herbal medicine. However, due to heterogeneity of studies, weak methodologies, and short-term follow-ups, the findings should be interpreted with caution.
